# Suboptimal states and frontoparietal network-centered incomplete compensation revealed by dynamic functional network connectivity in patients with post-stroke cognitive impairment

**DOI:** 10.3389/fnagi.2022.893297

**Published:** 2022-08-08

**Authors:** Bo Rao, Sirui Wang, Minhua Yu, Linglong Chen, Guofu Miao, Xiaoli Zhou, Hong Zhou, Weijing Liao, Haibo Xu

**Affiliations:** ^1^Department of Radiology, Zhongnan Hospital of Wuhan University, Wuhan, China; ^2^Department of Rehabilitation Medicine, Zhongnan Hospital of Wuhan University, Wuhan, China

**Keywords:** post-stroke cognitive impairment, resting-state functional magnetic resonance imaging, dynamic functional network connectivity, graph theoretic analysis, frontoparietal network

## Abstract

**Background:**

Neural reorganization occurs after a stroke, and dynamic functional network connectivity (dFNC) pattern is associated with cognition. We hypothesized that dFNC alterations resulted from neural reorganization in post-stroke cognitive impairment (PSCI) patients, and specific dFNC patterns characterized different pathological types of PSCI.

**Methods:**

Resting-state fMRI data were collected from 16 PSCI patients with hemorrhagic stroke (hPSCI group), 21 PSCI patients with ischemic stroke (iPSCI group), and 21 healthy controls (HC). We performed the dFNC analysis for the dynamic connectivity states, together with their topological and temporal features.

**Results:**

We identified 10 resting-state networks (RSNs), and the dFNCs could be clustered into four reoccurring states (modular, regional, sparse, and strong). Compared with HC, the hPSCI and iPSCI patients showed lower standard deviation (SD) and coefficient of variation (CV) in the regional and modular states, respectively (*p* < 0.05). Reduced connectivities within the primary network (visual, auditory, and sensorimotor networks) and between the primary and high-order cognitive control domains were observed (*p* < 0.01).

**Conclusion:**

The transition trend to suboptimal states may play a compensatory role in patients with PSCI through redundancy networks. The reduced exploratory capacity (SD and CV) in different suboptimal states characterized cognitive impairment and pathological types of PSCI. The functional disconnection between the primary and high-order cognitive control network and the frontoparietal network centered (FPN-centered) incomplete compensation may be the pathological mechanism of PSCI. These results emphasize the flexibility of neural reorganization during self-repair.

## Introduction

Stroke is the second leading cause of death in the world and a leading cause of long-term disability (Collaborators GBDS, 2019). Stroke caused by limited cerebral blood flow, whether ischemic stroke or hemorrhagic stroke, seriously destroys the structural and functional integrity of the local and whole range (Carrera and Tononi, 2014; Ward, 2017). Once stroke occurs, neural plasticity and reorganization happen in the area of injury and in the distant compartment to compensate for the loss of specialized neural tissue and function (Jones, 2017). Post-stroke cognitive impairment (PSCI) is one of the common functional disorders after stroke, affecting up to 1/3 of post-stroke survivors (Mijajlovic et al., 2017). Symptoms of cognitive impairment are independent predictors of high-order functional impairment and community reintegration 2–3 years after stroke (Kapoor et al., 2019). Exploring the neural mechanism of cognitive impairment after stroke is beneficial to early diagnosis and early intervention.

Functional neuroimaging has made an essential contribution to revealing the neural mechanism after stroke. Resting-state functional magnetic resonance imaging (rs-fMRI) studies have indicated that the brain’s spontaneous neural activity and functional connectivity (FC) have changed in patients with cognitive impairment after stroke (Peng et al., 2016; Cai et al., 2021). Peng et al. (2016) found a significant decrease in regional homogeneity in the bilateral anterior cingulate cortex and left posterior cingulate cortex/precuneus in PSCI patients compared to healthy volunteers and post-stroke non-cognitive impairment patients. In particular, the functional connectivities in the default mode network may be related to the occurrence of cognitive impairment after stroke and cognitive recovery (Park et al., 2014; Jiang et al., 2018). However, previous studies used analytical approaches that assumed static connectivity during the whole duration of the MRI scan, which does not allow for a fine-grained temporal evaluation of the rs-fMRI signal.

Time-varying characteristics of FC in a conscious and task-free human brain can be analyzed by “dynamic” functional network connectivity (dFNC) using rs-fMRI data (Hutchison et al., 2013b; Allen et al., 2014). dFNC allows connectivity between brain areas to differ over brief periods, contrary to the assumption of “static” connectivity across the whole duration of a functional MRI scan. Some studies evaluated the dynamic connectivity using independent component analysis (ICA) combined with the sliding window method to identify spatial maps in the windowed blood oxygen level-dependent (BOLD) signal (Premi et al., 2019; Bonkhoff et al., 2020). dFNC analyses can be displayed as various “connectivity states” of the brain and transition trajectories between them by summarizing recurrent large-scale connectivity patterns (Leonardi et al., 2014). Changes in dFNC have been linked to cognitive function, such as Alzheimer’s disease (Schumacher et al., 2019), Parkinson’s disease with dementia (Fiorenzato et al., 2019), mild cognitive impairment (Jiao et al., 2021), and subjective cognitive decline (Chen et al., 2021). Furthermore, the topological properties of the connectivity states and the meaning of the states can be illuminated by graph theory, which is an analysis method unraveling the complex network organization (Yu et al., 2018). Recently, accumulating evidence has found that stroke mainly leads to changes in the motor large-scale network’s FC and temporal properties (Bonkhoff et al., 2020; Wang et al., 2020). As shown in stroke patients, abnormal dynamic connectivity patterns could predict acute motor impairment and recovery (Bonkhoff et al., 2021). However, whether changes in dFNC are related to cognitive impairment after stroke is unclear.

This study aimed to estimate distinct dynamic connectivity states and their topological network properties to elaborate on the states’ meaning. Furthermore, we investigated the differences in dynamic connectivity among different PSCI groups and the healthy control group. We hypothesized that dFNC alterations resulted from neural reorganization in PSCI patients, and specific dFNC patterns characterized different pathological types of PSCI.

## Materials and methods

### Participants

This study included 37 PSCI patients and 21 healthy controls (HC) closely matched with the patient groups by age and gender. According to the age distribution of all patients, 21 intervals were divided and the mean age within the interval was calculated. The HC group was age-matched by adding or subtracting 4 years. We recruited PSCI patients from the Department of Neurological Rehabilitation at Zhongnan Hospital of Wuhan University, including 16 PSCI patients with hemorrhagic stroke (hPSCI group) and 21 PSCI patients with ischemic stroke (iPSCI group). Stroke patients were enrolled in this study if they had the symptoms of stroke, confirmed by CT or MRI (Yew and Cheng, 2015). Further inclusion criteria were: (1) first-ever stroke, 7 days–3 months; (2) 40–80 years old (Jiang et al., 2018); (3) at least one cognitive domain was impaired, the Montreal Cognitive Assessment scores (MoCA) was lower than 26 (Yin et al., 2020); (4) right-handed; and (5) voluntarily participated in this study and signed the informed consent form. Exclusion criteria were: (1) unstable vital signs; (2) postoperative craniotomy or skull defect; (3) pre-stroke cognitive impairment such as Alzheimer’s disease, Parkinson’s disease, dementia, and mild cognitive impairment; (4) severe aphasia or other reason that could not complete the cognitive test; (5) disorders that may interfere with cognitive assessment, such as mental illness; and (6) contraindications for MRI scanning. The inclusion criteria for HC were: (1) matched with the patient groups by age and gender; (2) the MoCA scores was higher than 26; (3) right-handed; (4) voluntarily participated in this study and signed the informed consent form. The exclusion criteria were same as the PSCI group.

Approval was obtained from the Medical Research Ethics Committee and Institutional Review Board of Zhongnan Hospital (2019012). The procedures used in this study adhere to the tenets of the Declaration of Helsinki. Informed consent was obtained from all individual participants included in the study.

### Behavioral assessment

A professional therapist conducted neuropsychological tests. The therapist has received systematic training on neuropsychological scale evaluation. All subjects underwent one neuropsychological test named the Beijing version of the Montreal Cognitive Assessment.

### Data acquisition and preprocessing

**FIGURE 1:**
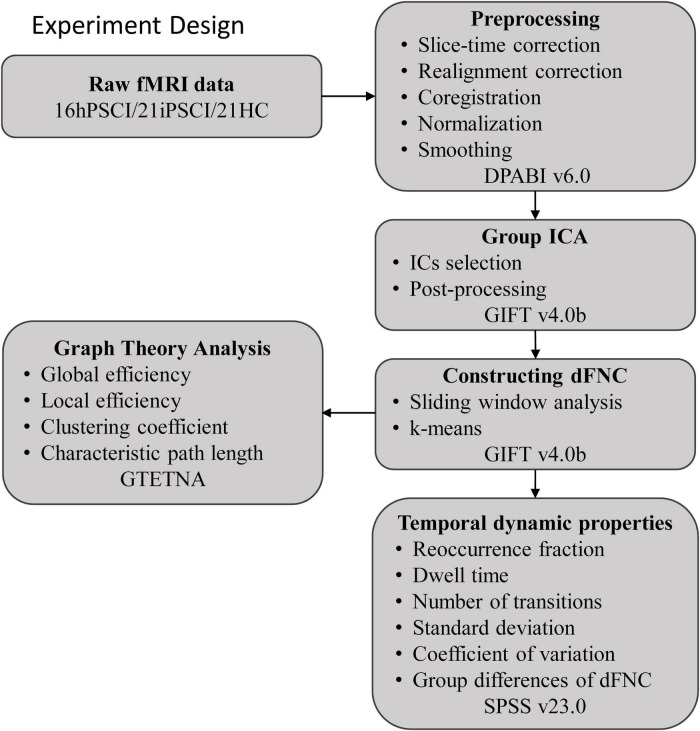
The entire experimental design processing flow. hPSCI, cognitive impairment group after hemorrhagic stroke; iPSCI, the cognitive impairment group after ischemic stroke; HC, health control group; ICA, independent component analysis; dFNC, dynamic functional network connectivity.

The entire experimental design processing flow is shown in Figure 1.

All images were acquired on a MAGNETOM Trio 3.0 T MR scanner (Siemens, Germany). Resting-state data were acquired using a gradient echo-planar imaging (EPI) sequence: TR/TE = 2,000/30 ms, FOV = 240 mm × 240 mm, flip angle (FA) = 78°, matrix = 64 × 64, thickness = 4.0 mm, number of slices = 35, and voxel size = 3.75 × 3.75 × 4 mm^3^. The number of acquired resting-state scans were 480 s. Structural 3D T1-weighted images were acquired with a three-dimensional magnetization-prepared rapid gradient echo (3D-MPRAGE) sequence: TR/TE = 2,000/2.3 ms, thickness = 1.0 mm, FA = 8°, FOV = 225 mm × 240 mm and voxel size = 1 × 1 × 1 mm^3^. During functional MRI scanning, participants were instructed to lie down in a comfortable position, close their eyes, stay awake and avoid thinking about anything if possible. Use two foam pads to fix the head to minimize the movement of the patient’s head and use the rubber earplugs to reduce the noise generated during the MRI scan to ensure the smooth progress of the MRI scan and the quality of the image.

Data preprocessing was performed in the MATLAB environment (version R2016b, Mathworks, Natick, MA, United States) using the SPM 12.^1^ Preprocessing procedures included the following steps: (1) the Neuroimaging Informatics Technology Initiative (NIFTI) format conversion from the raw DICOM images; (2) remove the first 10 time points to ensure steady-state magnetization; (3) the slice timing correction and realignment correction for head motion; (4) the coregistration of the structural and functional images; (5) normalized into the standard Montreal Neurological Institute (MNI) space with 3 × 3 × 3 mm^3^ resample; (6) the smoothing with a 4 × 4 × 4 mm^3^ full-width-half-maximum (FWHM) Gaussian kernel; Since excessive head motion can seriously affect the dFNC analysis (Hutchison et al., 2013a), we calculated individual maximum displacement and mean framewise displacement (FD) to reduce the potential head motion bias. We excluded participants with a maximum displacement above 2 mm and a maximum rotation above 2 degrees. We also excluded subjects with a mean FD > 0.5 mm (Power et al., 2012). According to these criteria, no subjects was excluded. The FD did not differ significantly among the three groups (*p* = 0.37). Our study finally enrolled 37 PSCI patients and 21 HC.

### Group independent component analysis

After preprocessing, all resting-state data were processed using the fMRI toolbox software (GIFT version 4.0b)^2^ to decompose the preprocessed images into different functional networks (Erhardt et al., 2011). Firstly, the number of independent components (ICs) was estimated using the minimum description length (MDL) based on all fMRI data, yielding 47 components. The data of all subjects were then reduced in dimensionality automatically using principal component analysis at the group level (Bell and Sejnowski, 1995). Secondly, the infomax ICA algorithm was repeated 30 times in ICASSO to ensure stability and reliability (Li et al., 2007). Thirdly, the data were back-reconstructed using the GICA algorithm to obtain object-specific spatial maps and time courses (Calhoun et al., 2001). Finally, we needed to identify ICs reflected the actual neural activity in the brain. The selection criteria were that the activation peak coordinates were predominantly in the gray matter with low spatial overlap with known vascular, ventricular, motion-related, and sensitivity artifacts (Cordes et al., 2000). In addition, the power of the corresponding time course signals should be predominantly concentrated in the low-frequency band, with the ratio of power below 0.10 and 0.15–0.25 Hz. Finally, 21 ICs were considered meaningful according to the above criteria, sorting into 10 resting-state networks (RSNs) based on the spatial correlation values between ICs and prior knowledge from previous studies (Shirer et al., 2012; Allen et al., 2014).

### Post-processing of time courses

After ICs selection, we performed additional post-processing steps on the 21 ICs using the published formula of Allen et al. (2014). Firstly, detrending the time course considering the data’s linear, quadratic, and cubic trends. Secondly, the effect of head motion was regressed to obtain more accurate results. Then, filtering with a fifth-order Butterworth low-pass filter with a high-frequency cutoff frequency of 0.15 Hz and removing spikes to ensure that artificial spikes would not negatively affect the signal analysis.

### Dynamic functional network connectivity

This study used the sliding window approach in the dFNC toolbox of GIFT to segment the time series of post-processed ICs to capture the dynamic changes of FC (Allen et al., 2014; Damaraju et al., 2014). By adding a rectangular window of length 22-TRs (corresponded to 44 s) with a Gaussian value of 3 TRs and a sliding step of 1TR to a BOLD time series of length 230-TRs, the entire scanned time series can be partitioned into 208 windows. We chose the window length of 22-TR based on Allen’s study (Allen et al., 2014), and cognitive states seemed to be identified at window length around 30–60 s (Shirer et al., 2012). We first estimated the regularized accuracy matrix (i.e., the inverse of the covariance matrix) to estimate the covariance matrix better (Smith et al., 2011). The penalty on the L1 norm was used in the graph LASSO framework to ensure sparsity, and the regularization parameters for each subject were optimized using the cross-again validation framework (Friedman et al., 2008). We regressed age, sex, mean framewise translation, and rotation covariates. Finally, we performed Fisher’s r-z transformation on all FC matrices. Thus, we obtained 208 FC matrices with 210 (21 × 20/2) edges for each subject, reflecting the time-varying FC pattern over the entire scan period.

After the sliding window analysis, we used the k-means clustering algorithm to determine the FC states by clustering the covariance matrix of all windows in all subjects (Lloyd, 1982). We used the squared Euclidean distance to estimate the similarity between window FC matrices. Using cluster number validity analysis, we evaluated the optimal number of clusters, computing as the ratio between the within-cluster and between-cluster distances. Finally, we determined that the optimal number of clusters was equal to four.

### Graph-theory parameter analysis

The GRETNA software^3^ was used to determine the topologic organization of each dFNC state based on the ICs resulting from the ICA analysis (Wang et al., 2015). We defined the 21 ICs and inter-21 correlations as nodes and edges, respectively. First, four FC matrices were fully connected with a sparsity threshold S, defined as the ratio of the number of actual edges to the maximum potential number of edges in a network. We set sparsity threshold values with a range of 0.05–0.5 in a step of 0.01 (Zhu et al., 2021). As a result, a weighted, undirected graph was produced. Then, calculate the topologic organization (Global efficiency, Local efficiency, Clustering coefficient, and Characteristic path length) of the network at each sparsity threshold. For each state, the area under the curve (AUC) was calculated for each topological metric over the entire threshold range to avoid the specific selection of a threshold (Koshimori et al., 2016).

### Dynamic properties analysis: Temporal properties and strength

We statistically evaluated the following dynamic connectivity measures of the dFNC states: (1) Reoccurrence fraction, which means the percentage of total time spent by subjects in a particular state; (2) Dwell time, which means time spent by subjects in a state without switching to another state; and (3) The number of transitions, which indicates how often subjects changed states.

We also calculated the correlation coefficient’s standard deviation (SD) and coefficient of variation (CV) across windows to scrutinize the variability of FC. SD is the standard deviation value of the FNC for all windows in each state, and CV is the ratio of SD to the mean of the FNC in time (Tijhuis et al., 2021). We calculated these two indexes in each state and the medians of the SD and CV (210 FNCs).

Additionally, we tested for group differences in dynamically connectivity pairs in each connection state.

### Statistical analysis

We analyzed general demographics and clinical variables using SPSS 23.0 software. First, the Shapiro-Wilk (S-W) test was applied for the normality of the distribution of scale scores. A three-level one-way analysis of variance (ANOVA) with *post-hoc t*-tests was used to compare the three groups’ age, years of education, mean FD, and MoCA scores. The two-sample *t*-test was used to detect differences in the disease duration and lesion volume between the stroke groups. A chi-square test was performed for the comparison of gender, lesion side, and lesion location. The significance level was set at 0.05.

The fractional time, dwell time, the number of transitions, SD, and CV were calculated with the one-way ANOVA with *post hoc* analysis (*p* < 0.05). If there was no significant difference after one-way ANOVA, a two-sample independent *t*-test was performed (*p* < 0.05). We performed a three-level one-way ANOVA with a *post-hoc t*-test to test for the connectivity strength of each state among the three groups (*p* < 0.01, uncorrected). Age, sex, years of education, disease duration, and FD were included as covariates in all analyses. Finally, the correlations between the dFNC measures of those significant between the groups and MoCA scores were performed using Pearson correlation analysis (*p* < 0.05).

## Results

### Demographic and clinical results

As shown in Table 1, age, sex, and education level did not differ significantly among the three groups (*p* > 0.05). We found no significant difference in the disease duration and lesion side between the hPSCI and iPSCI groups (*p* > 0.05). The thalamus and basal ganglia were the primary damage sites in the hPSCI group, whereas cortical infarction occurred in the iPSCI group (*p* = 0.003). The iPSCI group showed higher lesion volume than the hPSCI patients (*p* = 0.004). There were substantial differences in MoCA scores between the stroke and HC groups (*p* < 0.05). The hPSCI group had higher MoCA scores than the iPSCI group, although without significant difference (*p* > 0.05). The lesion overlaps for the two stroke groups were shown in Supplementary Figure 1.

**TABLE 1:** Demographic, clinical, and neuropsychological data in cognitive impairment group after hemorrhagic stroke (hPSCI group), the cognitive impairment group after ischemic stroke (iPSCI group), and healthy control group (HC group).

Variable	hPSCI (*n* = 16)	iPSCI (*n* = 21)	HC (*n* = 21)	*P*-value
Age (years)	60.38 ± 9.78	55.81 ± 10.61	60.10 ± 6.59	0.22
Sex (male/female)	12/4	18/3	17/4	0.57
Education (years)	11.31 ± 3.22	12.24 ± 3.03	11.81 ± 2.80	0.65
Disease duration (days)	49.93 ± 22.80	47.57 ± 50.00	−	0.87
Lesion side (Left/Right)	10/6	16/5	−	0.48
Lesion location (Cortex/Subcortex)	3/13	15/6	−	0.003
Lesion volume (Voxel)	25,027 ± 32,775	83,149 ± 69,981	−	0.004
MoCA	16.62 ± 4.57^a^	15.48 ± 5.22^a^	29.10 ± 1.14	<0.001

One-way ANOVA with post hoc test was used for age, years of education, MOCA scores among three groups; Two sample t-test was used for the duration of the disease and lesion volume; A Chi-square test was conducted to compare gender, lesion side, and lesion location; MoCA, Montreal Cognitive Assessment Scale.

^a^Significant compared to HC.

### Identification of resting-state networks

**FIGURE 2:**
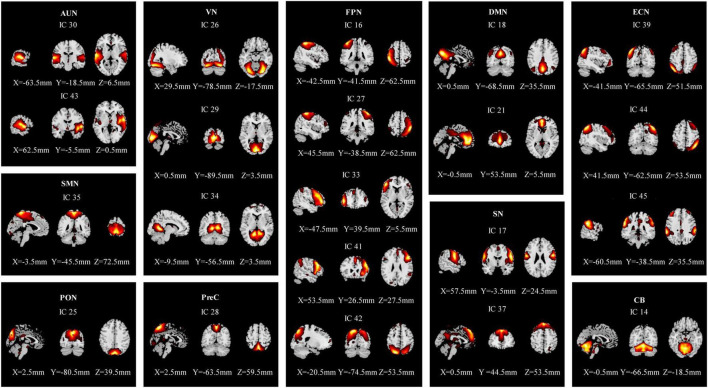
Spatial maps of the 21 independent network components. They were divided into 10 functional networks based on their anatomical and functional properties, including the auditory network (AUN), the visual network (VS), the sensorimotor network (SMN), the occipital network (PON), the precuneus network (PreC), the default mode network (DMN), the frontoparietal network (FPN), the executive control network (ECN), the salience network (SN), and the cerebellar network (CB).

The rs-fMRI data of 58 participants were successfully decomposed into 47 ICs, and the ICASSO returned a stability index of 0.96 (SD = 0.037), demonstrating high reliability. Finally, 21 ICs reflected the actual neural activity in the brain were identified. We identified 10 RSNs from the 21 ICs based on their anatomical and presumed functional properties (Damoiseaux et al., 2006; Smith et al., 2009; Zuo et al., 2010; Mueller et al., 2014; Xiao et al., 2017), including the auditory network (AUN: ICs 30, 43), the visual network (VN: ICs 26, 29, 34), the sensorimotor network (SMN: IC 35), the occipital network (PON: IC 25), the precuneus network (PreC: IC 28), the default mode networks (DMN: ICs 18, 21), the frontoparietal network (FPN: ICs 16, 27, 33, 41, 42), the executive control network (ECN: ICs 39, 44, 45), the salience network (SN: ICs 17, 37), and the cerebellar network (CB: IC 14) (Figure 2). The detailed information is shown in Supplementary Table 1.

### Dynamic functional network connectivity states analysis

**FIGURE 3:**
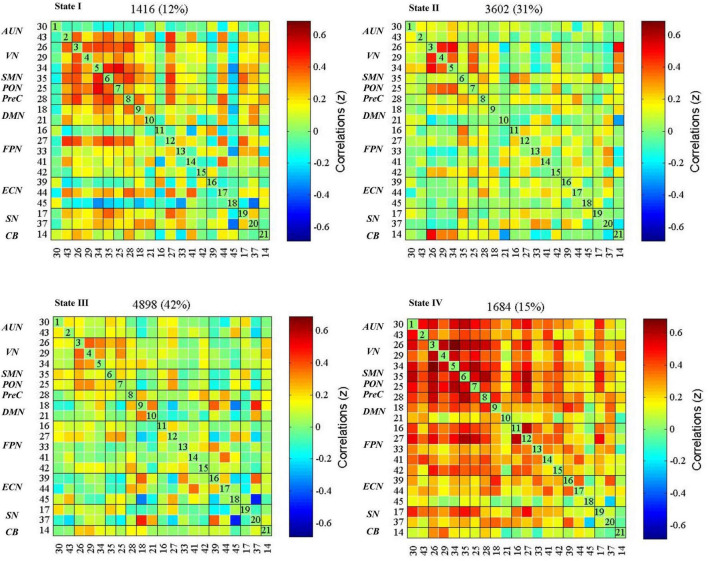
dFNC states in the whole sample (*k* = 4). The centroid of each functional network connectivity state with the total number of occurrences of each state and percentage of the total number of occurrences. The color bar represents the value of the correlations: Red color represents positive correlations and blue color represents negative correlations. AUN, the auditory network; VN, the visual network; SMN, the sensorimotor network; PON, the occipital network; PreC, the precuneus network; DMN, the default mode networks; FPN, the frontoparietal network; ECN, the executive control network; SN, the salience network; CB, the cerebellar network.

The dynamic interactions of the 10 functional networks were evaluated using the sliding window and *k*-means clustering analyses. The whole sample showed four reoccurring patterns during the rs-fMRI acquisition (Figure 3). State I (12% of all windows), a modular connectivity state, was characterized by high positive connections within primary perceptional domains. The primary network is the general term of visual network, auditory network and sensorimotor network (AUN, VN, SMN). State II (31% of all windows), a regional connectivity state, was characterized by the regional connection between the networks. State III (42% of all windows), a spare connectivity state, was generally characterized by extensively sparse FNCs. State IV (15% of all windows), a strong connectivity state, was described by tightly positive FNCs within and between all RSNs.

### Graph topological parameters

The global metrics of global efficiency, local efficiency, clustering coefficient, and characteristic path length were depicted in Supplementary Figure 2, calculated at each sparsity level. We found that state IV had the highest global efficiency, the lowest characteristic path length, the highest clustering coefficient, and local efficiency. State III showed the lowest clustering coefficient and local efficiency. These metrics for states I and II were between states III and IV (Table 2).

**TABLE 2:** The global properties for each state.

	State I	State II	State III	State IV
Global efficiency	0.0577743200	0.0489913187	0.0511881147	0.0891780906
Local efficiency	0.0814455927	0.0711789255	0.0680513656	0.1253151329
Clustering coefficient	0.1293941784	0.1148311677	0.1090590835	0.1546121243
Characteristic path length	4.0172062096	5.2242265733	4.7838186459	2.7482607561

### Dynamic properties analysis

**FIGURE 4:**
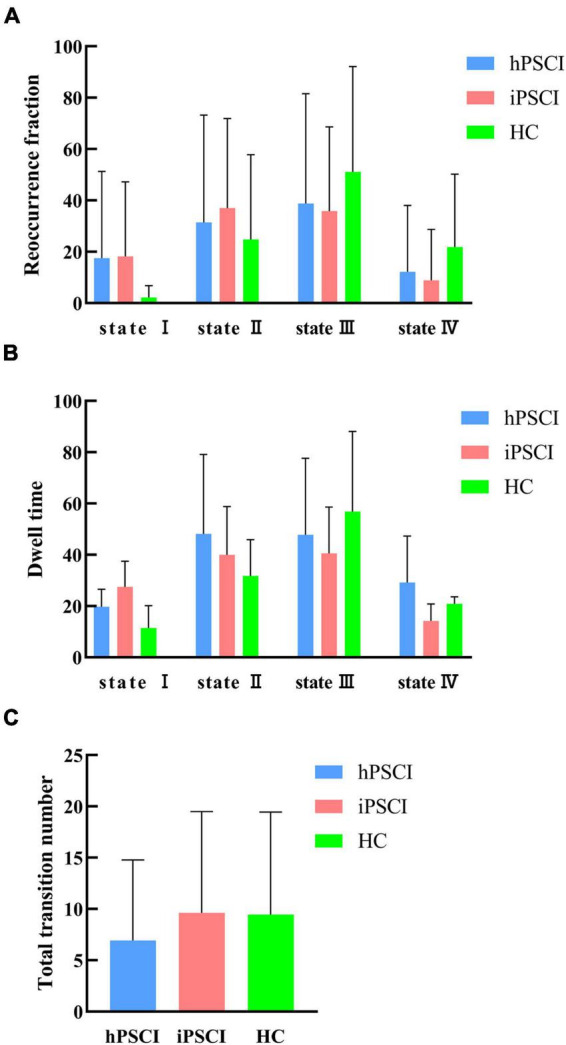
Temporal properties of dFNC states for the three groups. **(A)** Reoccurrence fraction; **(B)** dwell time; **(C)** number of transitions. The error bars represent standard deviation; hPSCI, cognitive impairment group after hemorrhagic stroke; iPSCI, the cognitive impairment group after ischemic stroke; HC, health control group.

Table 3 and Figure 4 showed that no significant differences in reoccurrence fraction, mean dwell time, or the number of transitions between states were found among the three groups (*p* > 0.05).

**TABLE 3:** The temporal properties for each state and each group.

Variables	hPSCI (*n* = 16)	iPSCI (*n* = 21)	HC (*n* = 21)	*F*	*P*-value
**State I**					
Reoccurrence fraction	17.53 ± 33.77	18.17 ± 29.08	2.19 ± 4.62	2.64	0.08
Dwell time	15.66 ± 27.56	22.30 ± 38.96	4.10 ± 9.18	2.25	0.12
**State II**					
Reoccurrence fraction	31.45 ± 41.76	37.02 ± 34.88	24.76 ± 33.01	0.60	0.55
Dwell time	50.51 ± 77.84	40.03 ± 58.71	26.59 ± 47.71	0.71	0.49
**State III**					
Reoccurrence fraction	38.81 ± 42.75	35.91 ± 32.74	51.14 ± 40.99	0.90	0.41
Dwell time	52.47 ± 75.00	44.12 ± 56.55	71.29 ± 78.26	0.82	0.45
**State IV**					
Reoccurrence fraction	12.19 ± 25.89	8.91 ± 19.82	21.91 ± 28.26	1.54	0.22
Dwell time	21.88 ± 49.84	7.95 ± 13.75	17.98 ± 23.55	1.04	0.34
Total transition number	2.38 ± 2.42	4.33 ± 3.54	3.86 ± 3.48	1.74	0.19

One-way ANOVA with post hoc test was used. hPSCI, cognitive impairment group after hemorrhagic stroke; iPSCI, the cognitive impairment group after ischemic stroke; HC, health control group.

**FIGURE 5:**
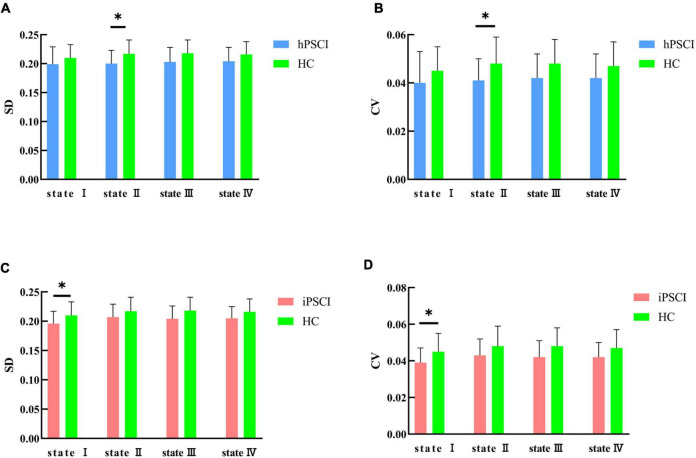
SD and CV differences between groups. **(A)** SD difference between hPSCI patients and HC; **(B)** CV difference between hPSCI patients and HC; **(C)** SD difference between iPSCI patients and HC; **(D)** CV difference between iPSCI patients and HC. SD, standard deviation; CV, coefficient of variation; hPSCI, cognitive impairment group after hemorrhagic stroke; iPSCI, the cognitive impairment group after ischemic stroke; HC, health control group; *P < 0.05. The error bars represent standard deviation of SD and CV.

We discovered that hPSCI patients showed lower whole-brain SD and CV in state II compared with HC (SD: *t* = 2.17, *p* = 0.037; CV: *t* = 2.07, *p* = 0.046) (Figures 5A,B), while iPSCI patients showed lower whole-brain SD and CV in the state I compared with HC (SD: *t* = 2.06, *p* = 0.046; CV: *t* = 2.15, *p* = 0.038) (Figures 5C,D).

**FIGURE 6:**
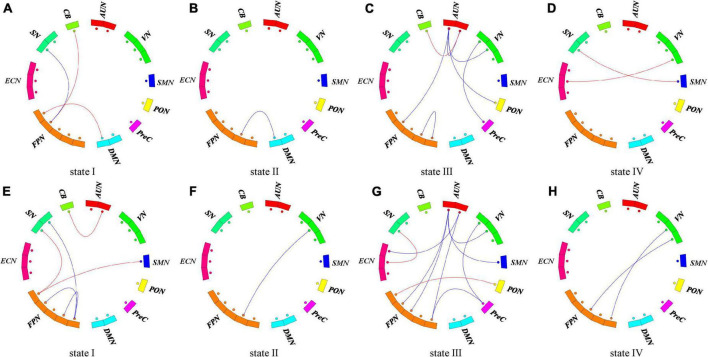
Functional connectivity differences in each state. **(A–D)** The difference of functional connection between hPSCI and HC groups in each state. **(E–H)** The difference of functional connection between iPSCI and HC groups in each state. *P* < 0.01, uncorrected. The red lines represent stronger functional connectivity than the HC group. The blue lines represent weaker functional connectivity than the HC group.

Additionally, we compared the strength of connections in the stroke groups and HC group in each state (Figure 6). Both PSCI patients showed more reduced FNC in state III, and increased FNC between FPN and other higher-order cognitive networks and primary networks compared with the HC. We observed two weaker within-network connections (FPN, AUN in state III), six weaker between-network connections (state I: FPN-SN; state II: FPN-DMN; state III: AUN-VN, AUN-PON, AUN-FPN, VN-PreC) and five stronger between-network connection (state I: FPN-DMN, FPN-CB; state III: AUN-CB; state IV: SN-SMN, ECN-VN) in the hPSCI group compared to the HC group (Figures 6A–D). We also observed 12 weaker between-network connections in the iPSCI group compared to HC (state I: FPN-SN; state II: FPN-VN; state III: AUN-VN, AUN-SMN, AUN-FPN, AUN-ECN, VN-PreC, FPN-PreC; state IV: FPN-VN), while two within-network were also weaker in state I (FPN). We found that the iPSCI group had five stronger between-network connections than the HC group (state I: AUN-CB, FPN-SMN, FPN-SN; state III: FPN-PON, ECN-SN) (Figures 6E–H) (*p* < 0.01, uncorrected). No significant FNC differences were found between the hPSCI and iPSCI groups. No significant correlations were found between the dFNC measures of those significant and MoCA scores.

## Discussion

This study was the first to use the dFNC method and graph theory analysis to estimate distinct dynamic connectivity states and their topological network properties to elaborate on the meaning of state. We identified 10 resting-state networks, and the dFNCs in the whole brain network can be clustered into four reoccurring states (modular state, regional state, sparse state, and strong state). For reoccurrence fraction and mean dwell time, there was an increasing trend in modular and regional states and a decrease in sparse and strong states in the PSCI groups. The hPSCI patients with the main thalamus and basal ganglia injury sites exhibited lower SD and CV in the regional state than HC. In contrast, the iPSCI patients with predominantly cortical infarcts showed lower SD and CV in the modular state. Compared to HC, reduced connectivities within the primary network (AUN, VN, SMN) and between the primary and high-order cognitive control domains were observed, especially FPN-centered FNC.

The dFNCs in the whole brain network could be clustered into four reoccurring states (state I-IV: modular connectivity state, regional connectivity state, sparse connectivity state, and strong connectivity state). Wang et al. (2020) also clustered patients with chronic-phase pontine infarction into four reoccurring states using dFNC. They found an imbalance in the separation and integration of whole-brain functions (Wang et al., 2020). Unlike other studies, we calculated the topological properties of each state to understand the meaning of states. We found that state IV had the highest global efficiency and the lowest characteristic path length among the four states, exhibiting extensive tight connectivity within and among all RSNs. The global efficiency and characteristic path length are well-known indicators of integration defined as the ability to rapidly combine specialized information from distributed brain regions (Rubinov and Sporns, 2010; Shine et al., 2016). State III had lower global efficiencies and higher characteristic path lengths, suggesting the information integration efficiency of state III was not as strong as that of state IV. In addition, state IV showed the highest clustering coefficient and local efficiency among all four states, implying the strongest functional segregation. The clustering coefficient indicates the degree of network grouping, and a higher clustering coefficient means a higher degree of network modularity (Cohen and D’Esposito, 2016). The local efficiency reflects the information transferability of local neighboring brain regions (Rubinov and Sporns, 2010). Thus, state IV was a highly segregated state, and state III was the least segregated state; states I and II were intermediate. In conclusion, state IV was a strong state with the highest network integration and segregation; state III was a sparse state with the least segregation ability (Wang et al., 2020). The degree of network integration and segregation of states I and II were between states III and IV; hence, we refer to states I and II as suboptimal states based on the topological properties, showing the network’s redundancy (Yamashita et al., 2021).

This study found that the three groups’ differences in reoccurrence fraction and dwell time in all connection states did not reach statistical significance. Compared with the HC group, there was an increasing trend for reoccurrence fraction and dwell time in states I and II and a decreasing trend for reoccurrence fraction and dwell time in states III and IV in the PSCI group. We discreetly speculate that the damage of regional neural activity and the disruption of function network integrity caused by stroke could be responsible for the decreasing trend in reoccurrence fraction and dwell time in states III and IV in PSCI patients. At the same time, to compensate for the lost function, the brain compensates toward the suboptimal states, i.e., state I and state II. Patients with PSCI exhibit overall cognitive, visual, and language dysfunctions, which may be caused by the disruption of information exchange between subcortical tissues and cognitively relevant brain areas (Smith, 2017). It has been demonstrated that the brain has the ability to self-repair to adapt to pathological changes following injury (Marlier et al., 2015; Szelenberger et al., 2020). The trend of increased states I and II may be a compensatory mechanism to compensate for the severe deficits in some cognitive functions during subacute phase. We speculated that the suboptimal states showed that network redundancy played an essential role in cognitive self-recovery in patients with PSCI.

In addition, we found that hPSCI patients showed lower whole-brain SD and CV in state II, and iPSCI patients showed lower whole-brain SD and CV in the state I compared with HC. The variability of dFNC measures means the ability of exploring cognition. High variability of dFNC measures means high-order brain areas, and low variability of dFNC measures means low-order brain areas. One study revealed functional organization was more variable in the high-order areas than in primary areas such as sensory/perceptual auditory processing (Tahmasebi et al., 2012). Hu et al. (2018) found that stroke patients exhibited reduced regional, temporal variability in the acute phase and gradually recovered over time. In particular, increased temporal variability in the ipsilesional precentral gyrus correlated with motor recovery (Hu et al., 2018). Low SD and CV mean the decreased exploratory capacity between the RSNs (Tahmasebi et al., 2012; Jalilianhasanpour et al., 2021). Our results suggested that cognitive impairment may be associated with the decline in SD and CV. We speculated that even though modular and regional states were compensating, they had not yet returned to normal levels and remained in the decompensation situation during the subacute phase in iPSCI patients.

Furthermore, the decrease of SD and CV in different types of stroke occurred in different states, which may be related to the location and pathological of the lesion. In the hPSCI group, the damage site was mainly subcortical, with the thalamus and basal ganglia predominant. A hemorrhagic stroke occurs when a blood vessel ruptures, causing the blood supply to neurons disrupted, mechanical tissue rupture, and hematoma formation (Xiao et al., 2017). The basal ganglia and thalamus are considered to be essential sensory conduction relay stations that are linked to perceptual, cognitive, and motor processes (Moustafa et al., 2017). They had extensive connections with the cerebellum and the cerebral cortex, but they were slightly injured, not completely destroyed, which may cause decreased SD and CV in the regional state (Bostan and Strick, 2018). In the iPSCI group, cortical infarction caused by the middle cerebral artery’s injury was predominant. Network damage after an ischemic stroke occurs in the local vicinity of the lesion and ischemic penumbra, which may be related to the decreased SD and CV in a modular state characterized by high positive connections within primary perceptional domains (Sekerdag et al., 2018). The location and severity of the injury may be related to the damage of SD and CV, which may influence the prognosis of cognitive impairment. Future studies should further investigate the SD and CV differences in different states based on the location of the injury in a larger imaging dataset. Perhaps in the future, we can analyze the prognosis of stroke patients by using different states combined with SD and CV analysis through longitudinal MRI data from a large sample of stroke patients.

We also investigated FNC differences between groups in each state. Both PSCI patients in this study showed more reduced FNC in state III compared with the HC. They showed reduced network connectivity within the primary domains and between the primary and high-order cognitive control domains, indicating internal and external damage. The iPSCI group manifested more disconnection between the primary and high-order cognitive control domains than the hPSCI group, consistent with the lower cognitive scores in the iPSCI group compared with the hPSCI group. This may be due to ischemia leading to more apoptosis and neuronal necrosis. In particular, the functional network connectivity centered on FPN was impaired. Most previous studies have found that abnormalities in the DMN and ECN increase the risk of PSCI using static FNC (Ding et al., 2014; Lim et al., 2014; Siegel et al., 2016). Our study suggested that damage was FPN-centered, suggesting dynamic FNC illuminate different mechanisms. Many studies have reported that FPN is related to cognitive control and episodic memory (Kompus et al., 2009; Ray et al., 2020). Our study suggested that the functional disconnection between the primary and FPN-centered high-order cognitive control network may lead to cognitive impairment after stroke.

In addition, our results showed increased FNC between FPN and other higher-order cognitive networks and primary networks in both PSCI groups. The brain can adapt to pathological changes by enabling and developing compensatory networks due to the impairment of some circuits (Barulli and Stern, 2013). Studies found that the area surrounding the stroke injury area formed a new connection with distant brain areas to compensate for the dysfunction brought by some damaged brain areas (Brown et al., 2007; Zhao et al., 2021). Zhao et al. (2018) indicated enhanced connectivity between the right FPN and DMN, hypothesizing that it might be a compensatory reorganization for the reduced inter−network connectivity between the posterior DMN and VN. Our results indicated that increased FPN-centered FNC might compensate for maintaining the cognitive load after the onset of cognitive impairment. We inferred that cognitive impairment resulted from FPN-centered FC compromised and incomplete compensation.

The limitations of this study were: (1) The small sample size and the significant individual differences. The large variance in making comparisons between the three groups may lead to some present trends but not significant. Although there were no significant differences in our study, the results of mean trends in small samples should be of equal concern. In addition, we should divide patients into subsamples of cortical lesions or subcortical lesions to further study the effects of lesion location; (2) No longitudinal study was conducted. It is unclear how these changes changed with stroke recovery. A longitudinal follow-up study would be performed in the near future; (3) There is no fixed standard for the length of the sliding window, and the length of 22TR was chosen in our study based on experience. We can try different window lengths in the future; (4) k-mean is the most used clustering method, characterized by no overlap between clustering centers of mass, and other clustering methods can be tried; (5) The results related to group-comparison of the connectivity strength could be corrected by multiple-comparison such as FDR to get more accurate results, and (6) After one-way ANOVA, there was no significant difference among three groups for SD and CV indices. Then, we used the two-sample *t*-tests to observe the between-group difference (hPSCI vs. HC, iPSCI vs. HC). This is an uncommon procedure because *post hoc* tests are only performed if the initial global test is significant. Thus, we should increase the sample size and do further research using one-way ANOVA with *post hoc* tests in the future.

## Conclusion

In this study, we explored the temporal characteristics of different types of PSCI based on dFNC analysis. There was a tendency to transition toward suboptimal states in patients with PSCI, and the redundancy of the suboptimal states may play a compensatory role. The reduced exploratory capacity in different suboptimal states characterized cognitive impairment and pathological types of PSCI. The functional disconnection between the primary and FPN-centered high-order cognitive control network may lead to cognitive impairment after stroke. Increased FPN-centered FNC might compensate for maintaining the cognition function. These results emphasize the flexibility of neural reorganization during self-repair.

## Data availability statement

The original contributions presented in this study are included in the article/Supplementary material, further inquiries can be directed to the corresponding author/s.

## Ethics statement

Approval was obtained from the Medical Research Ethics Committee and Institutional Review Board of Zhongnan Hospital (2019012). The patients/participants provided their written informed consent to participate in this study.

## Author contributions

BR: conceptualization, methodology, writing—review and editing, and funding acquisition. SW: conceptualization, formal analysis, and writing—original draft preparation. MY: conceptualization, formal analysis, and writing—review and editing. LC: writing—review and editing. GM and XZ: data collection. HZ: formal analysis. WL and HX: conceptualization, funding acquisition, and supervision. All authors listed have made a substantial contribution to the work and approved it for publication.

## Footnotes

1
http://www.fil.ion.ucl.ac.uk/spm

2
http://icatb.sourceforge.net

3
http://www.nitrc.org/projects/gretna
