# Supplementary material for: Suboptimal states and frontoparietal network-centered incomplete compensation revealed by dynamic functional network connectivity in patients with post-stroke cognitive impairment
Source: Front Aging Neurosci. 2022 Aug 8;14:893297. doi: 10.3389/fnagi.2022.893297 (PMC9393744; doi:10.3389/fnagi.2022.893297)
Supplement: Supplementary file 1 [file Data_Sheet_1.docx]

***Supplementary data***

Supplementary Table 1 Peak activation information of 21 independent components

| **RSN** | **IC** | ***Nv*** | ***T*_max_** | **Peak coordinate** | | | **Region** |
| --- | --- | --- | --- | --- | --- | --- | --- |
|  |  |  |  | **x** | **y** | **z** |  |
| **AUD** | IC 30 | 2345 | 9.220 | -63 | -18 | 6 | L Middle Temporal Gyrus |
|  |  |  |  |  |  |  | L Middle Temporal Gyrus |
|  |  |  |  |  |  |  | L Superior Temporal Gyrus |
|  |  |  |  |  |  |  | R Superior Temporal Gyrus |
|  |  |  |  |  |  |  | R Middle Temporal Gyrus |
|  |  |  |  |  |  |  | R Superior Temporal Gyrus |
|  | IC 43 | 2104 | 7.274 | 63 | -3 | 0 | R Superior Temporal Gyrus |
|  |  |  |  |  |  |  | R Superior Temporal Gyrus |
|  |  |  |  |  |  |  | R Temporal Pole |
| **VN** | IC 26 | 3081 | 9.405 | 30 | -78 | -18 | R Cerebelum (VI) |
|  |  |  |  |  |  |  | L Cerebelum (Crus 1) |
|  | IC 29 | 1905 | 10.937 | 0 | -90 | 3 | L Calcarine Gyrus |
|  | IC 34 | 2395 | 9.757 | -9 | -54 | 3 | L Linual Gyrus |
|  |  |  |  |  |  |  | R Linual Gyrus |
|  |  |  |  |  |  |  | L Fusiform Gyrus |
| **SMN** | IC 35 | 2579 | 10.252 | -3 | -45 | 72 | L Precuneus |
|  |  |  |  |  |  |  | R Postcentral Gyrus |
| **PoN** | IC 25 | 1718 | 13.368 | 3 | -81 | 39 | L Cuneus |
|  |  |  |  |  |  |  | R Linual Gyrus |
|  |  |  |  |  |  |  | L Linual Gyrus |
| **PreC** | IC 28 | 1334 | 15.539 | 3 | -66 | 57 | R Precuneus |
| **DMN** | IC 18 | 1878 | 12.739 | 0 | -72 | 39 | L Precuneus |
|  | IC21 | 2352 | 10.064 | 0 | 54 | 6 | L Superior Medial Gyrus |
|  |  |  |  |  |  |  | L Middle Frontal Gyrus |
| **FPN** | IC 16 | 2244 | 11.650 | -42 | -42 | 63 | L Postcentral Gyrus |
|  |  |  |  |  |  |  | L Precentral Gyrus |
|  |  |  |  |  |  |  | L Precentral Gyrus |
|  | IC27 | 2197 | 12.453 | 45 | -39 | 63 | R Postcentral Gyrus |
|  |  |  |  |  |  |  | R Precentral Gyrus |
|  |  |  |  |  |  |  | R Precentral Gyrus |
|  | IC 33 | 2019 | 8.472 | -48 | 39 | 6 | L IFG (p. Triangularis) |
|  |  |  |  |  |  |  | L IFG (p. Triangularis) |
|  |  |  |  |  |  |  | R IFG (p. Triangularis) |
|  | IC 41 | 2371 | 6.550 | 51 | 24 | 30 | R IFG (p. Triangularis) |
|  |  |  |  |  |  |  | R IFG (p. Orbitalis) |
|  |  |  |  |  |  |  | L Cerebelum (VI) |
|  |  |  |  |  |  |  | L Cerebelum (Crus 1) |
|  |  |  |  |  |  |  | L Cerebelum (IV-V) |
|  |  |  |  |  |  |  | L Superior Medial Gyrus |
|  | IC 42 | 1652 | 10.086 | -18 | -75 | 54 | L Superior Parietal Lobule |
|  |  |  |  |  |  |  | L Calcarine Gyrus |
|  |  |  |  |  |  |  | R Superior Occipital Gyrus |
| **ECN** | IC 39 | 1115 | 11.304 | -39 | -69 | 51 | L Angular Gyrus |
|  |  |  |  |  |  |  | L Middle Temporal Gyrus |
|  |  |  |  |  |  |  | R Inferior Parietal Lobule |
|  |  |  |  |  |  |  | L MCC |
|  | IC 44 | 1388 | 12.138 | 39 | -63 | 57 | R Angular Gyrus |
|  |  |  |  |  |  |  | R Precuneus |
|  |  |  |  |  |  |  | R Middle Frontal Gyrus |
|  |  |  |  |  |  |  | R Middle Frontal Gyrus |
|  | IC 45 | 747 | 7.040 | -60 | -39 | 36 | L Supra Marginal Gyrus |
|  |  |  |  |  |  |  | L Inferior Parietal Lobule |
|  |  |  |  |  |  |  | R Supra Marginal Gyrus |
|  |  |  |  |  |  |  | R Inferior Parietal Lobule |
|  |  |  |  |  |  |  | L Middle Frontal Gyrus |
|  |  |  |  |  |  |  | R Middle Frontal Gyrus |
| **SN** | IC 17 | 1385 | 9.304 | 57 | -3 | 24 | R Precentral Gyrus |
|  |  |  |  |  |  |  | R Middle Frontal Gyrus |
|  |  |  |  |  |  |  | R Insula Lobe |
|  |  |  |  |  |  |  | L Postcentral Gyrus |
|  |  |  |  |  |  |  | L Precentral Gyrus |
|  | IC 37 | 2023 | 10.616 | 0 | 45 | 51 | L Superior Medial Gyrus |
|  |  |  |  |  |  |  | L Middle Frontal Gyrus |
|  |  |  |  |  |  |  | R Middle Frontal Gyrus |
| **CB** | IC 14 | 3813 | 6.602 | 0 | -66 | -21 | Cerebellar Vermis (7) |
|  |  |  |  |  |  |  | L Cerebelum (Crus 1) |
|  |  |  |  |  |  |  | R Cerebelum (VI) |

Table shows all local maxima separated by more than 20 mm. Regions were automatically labeled using the Anatomy Toolbox atlas. The coordinates are peak voxel coordinates of the one-sample t-test results for each independent component spatial maps of all subjects. RSN: resting-state network; AUN: the auditory network; VN: the visual network; SMN: the sensorimotor network; PON: the occipital network; PreC: the precuneus network; DMN: the default mode networks; FPN: the frontoparietal network; ECN: the executive control network; SN: the salience network; CB: the cerebellar network; IFG: Inferior frontal gyrus; MCC: middle cingulate cortex; Nv: number of voxels in each cluster; R: right; L: left

Figure S1 The lesion overlaps for the two stroke groups. (A) The lesion overlaps for hPSCI group; (B) The lesion overlaps for iPSCI group. The color bar represents the probability of lesion.


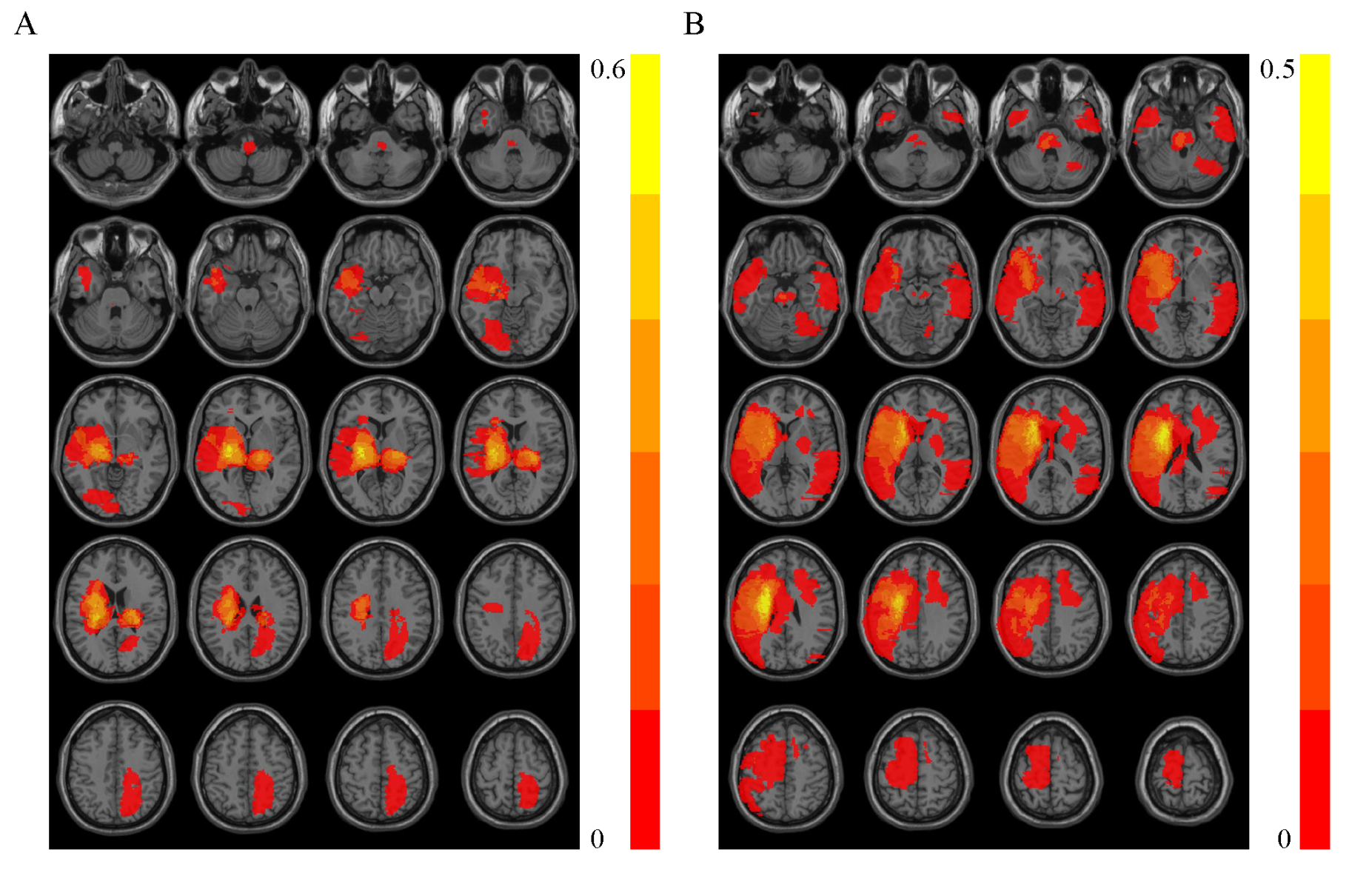


Figure S2 The global properties for each state at each sparsity level. (A) Global efficiency; (B) Local efficiency, (C) Clustering coefficient; (D) Characteristic path length
